# Comparative Evaluation of Salivary Enzyme in Patients With Gingivitis and Periodontitis: A Clinical-Biochemical Study

**DOI:** 10.7759/cureus.20991

**Published:** 2022-01-06

**Authors:** Khalid Alsaykhan, Nubesh S Khan, Mohammed I Aljumah, Abdulrahman S Albughaylil

**Affiliations:** 1 Dentistry, Qassim University, Arrass, SAU; 2 Periodontology, Qassim University, Arrass, SAU

**Keywords:** salivary enzyme, saliva, periodontitis, gingivitis, aspartate aminotransferase

## Abstract

Objective

This study aims to relate aspartate aminotransferase (AST) in saliva and periodontal status in patients with gingivitis and periodontitis.

Methods

Forty-five patients have undergone a periodontal detailed examination as well as indexes sorted and classified into three gingival-based groups: healthy, gingivitis and periodontitis. Fifteen (15) patients were assigned for each group. Ten milliliter of stimulated saliva from a patient was collected after rinsing the mouth with 15 mL of water in a sterile tube. Biochemical analysis was conducted using the study GOT (ASAT) IFCC mod. liquiUV kit from HUMAN. Kinetic method for the determination of GOT (ASAT) activity and TC 84 Teco diagnostics chemistry analyzer.

Result

Acquired results indicated statically significant increases of AST level in saliva from patients with periodontitis and gingivitis (p < 0.01) in relation to the control group.

Conclusion

These results revealed that salivary AST level is higher in patients that have periodontal destruction, pocket depth and bleeding in probing. This clinically indicated that salivary biomarkers can be used as a diagnostic tool for the evaluation of periodontal health status.

## Introduction

Periodontitis is a common inflammatory disease that has many causes. Periodontitis is mainly known as an inflammatory disease that affects the periodontium [1]. Periodontitis is caused mainly by microorganisms that adhere to teeth and grow. Periodontitis is diagnosed by probing the gingival sulcus and using a radiograph. Although for assessment and determination of the current status of the disease, these procedures are not good. But, they are excellent to assess and determine the disease history [2].

 More recent studies in periodontal diseases are focused on methods that can detect periodontal risk using biomechanical markers. These markers have an important role in the detection of early inflammatory changes in a short period of time [3]. Saliva has been used as a diagnostic fluid in dentistry. Salivary content for periodontal diagnosis includes bacteria and bacterial products, enzymes and immunoglobins, hormones of host origin, ions, and volatile compounds [4]. Damaged cells of periodontium lead to increasing the release of intracellular enzymes into the gingival crevicular fluid (GCF) and saliva [5]. Diagnosis of saliva is a developing field that has grown over many significant developments in the last decade. A lot of enzymes that are assessed for early diagnosis of periodontal disease are aspartate aminotransferase (AST) [6].

AST is an enzyme that can be found in cells all over the body. Although the liver and heart are the most organs that contain it [7], levels of AST in the blood are low in healthy individuals. However, when an organ such as the liver or the heart is injured or diseased, the AST blood level will increase. There exists a direct relation between AST level and quantity of tissue impairment [8].

Aim

The aim of the study is to evaluate salivary AST in patients with gingivitis and periodontitis.

Objectives

The objective is to estimate the levels of enzymes AST in the saliva of healthy subjects, gingivitis, and chronic periodontitis patients, to assess and compare the activity of the enzyme between healthy subjects, gingivitis and chronic periodontitis groups and to correlate the level of an estimated enzyme with that of clinical parameters in healthy subjects, gingivitis patients and patients with chronic periodontitis.

## Materials and methods

The clinical study was conducted at the Dental Clinic, Arrass Dental College, Qassim University, as a total number of 45 participants with the age range of 25-50 years were selected for the study and were divided into three groups of 15 each. As for Group I Periodontally healthy individuals (Control Group): it consisted of contributors with probing depth equal to or less than 3mm, at least 20 natural teeth and with no attachment loss and bleeding on probing with less than 20% sites. Group II Chronic gingivitis patients (Study Group): this group was composed of contributors with probing depth equal to or less than 3mm, at least 20 natural teeth and absence of attachment loss and bleeding on probing with more than 20% sites. Group III Generalized chronic periodontitis (Study Group): it was made up of contributors who should have five qualifying sites in two quadrants with a minimum of two affected teeth in each quadrant with each site having probing depth more than or equal 5mm, CAL more than or equal 3mm, bleeding on probing.

The nature of the study was informed to all the participants and an informed consent form was taken before the beginning of the study.

All subjects with no history of systemic disease and were in good general health were included in the study. Patients with a history of alcohol abuse and smoking as well as individuals who have undergone periodontal treatment in the past six months or taken antibiotics in the past six months were excluded. All female patients were also excluded from the study.

**Figure 1:**
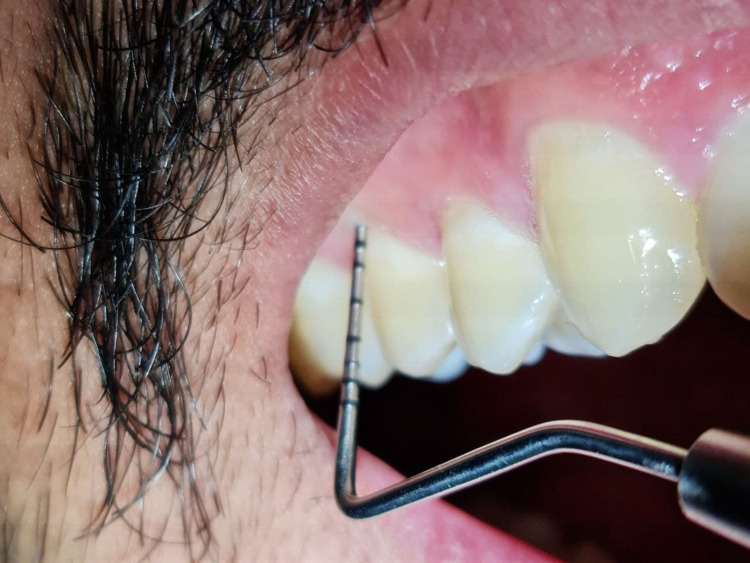
Measure probing depth using William's periodontal probe

At the initial examination, a detailed medical questionnaire was taken. Then, a complete periodontal examination, which included: Plaque Index (Silness and Loe), Gingival Bleeding Index (Loe and Silness), Probing Pocket Depth and Clinical Attachment Level, was conducted. Periodontal examinations were completed by dental undergraduates under the supervision of an assistant professor and periodontics specialist using William's periodontal probe (Figure 1) and mouth mirror.

All the participants in the study were asked to rinse with 15 mL of water (to wash out exfoliated cells). The stimulated saliva (10 mL) of the patients was collected in a sterile tube (13x75mm). After that, the saliva samples were sent to the laboratory immediately for analysis of AST.

**Figure 2:**
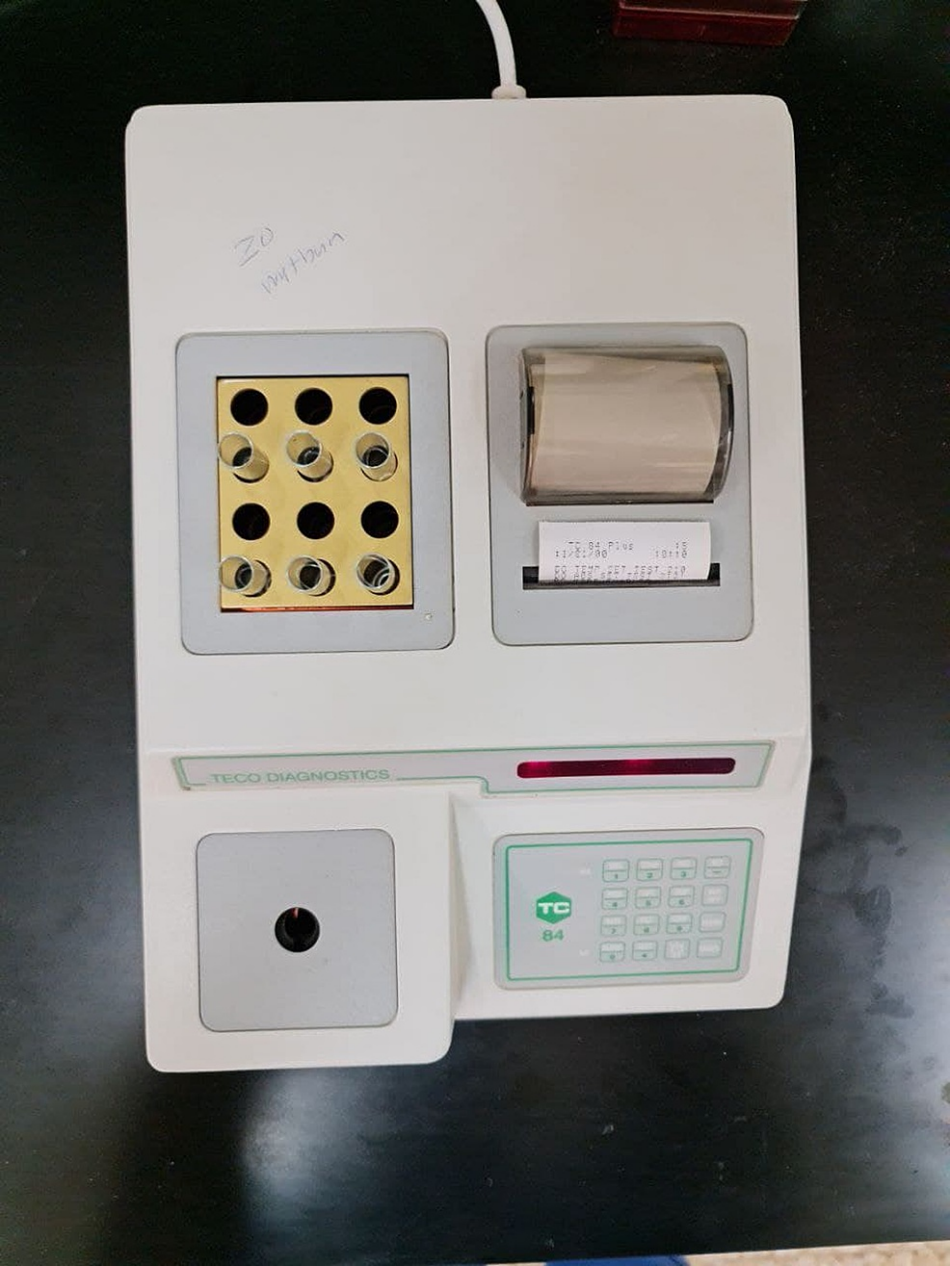
TC 84 Teco diagnostics chemistry analyzer

Biochemical analysis used TC 84 Teco diagnostics chemistry analyzer (Figure 2) and GOT (ASAT) IFCC mod. liquiUV kit from HUMAN. The kinetic method for the determination of GOT (ASAT) activity was utilized according to the recommendation of the Expert Panel of IFCC (International Federation of Clinical Chemistry and Laboratory Medicine) without pyridoxal phosphate activation.

Pipette 1,000µm of reagent was put into a glass test tube (12x100mm) then incubation for the desired temperature (37c) was applied. After it reached the desired temperature, it was mixed with 100 µm of saliva and incubated at the mixture at the desired temperature. The absorbance was read after 1 min and at the same time, the stopwatch was started. The absorbance was read again exactly after 1, 2 and 3 min.

Statistical analysis

Data were statistically analyzed using an unpaired t-test. SPSS version 26 (IBM, Armonk, NY, USA) and MS Excel were used to analyze the data.

Ethical approval and confidentiality

The data were collected anonymously and retained no identifying information. When the data were collected, we applied the inclusion and exclusion criteria. Then, the data were entered into an Excel file. Ethical approval was taken from Alrass Dental Research Center, Dental Ethical Committee Code: DRC/16M/4-20.

## Results

A total of 45 subjects were divided into three groups consisting of periodontally healthy patients (n=15), gingivitis patients (n=15) and chronic periodontitis patients (n=15).

**Figure 3:**
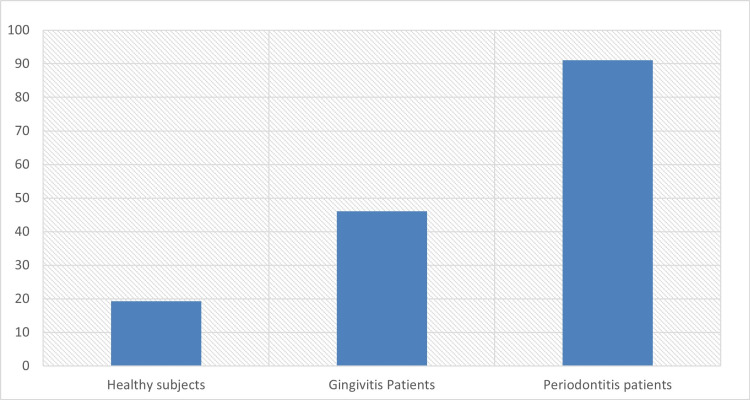
A comparison of mean salivary AST between three groups AST - aspartate aminotransferase

Median levels of salivary AST and for the three groups are reported in Table 1 and Figure 3.

**Table 1:** Median and interquartile range of AST levels for study groups AST - aspartate aminotransferase

Group	Median	Interquartile range
Healthy	19.29	15
Gingivitis	46.07	23.57
Periodontitis	91.08	38.57

Statistical analysis revealed significant (t = 6.73, p < 0.01) differences between salivary AST levels in patients with gingivitis compared with the healthy group as shown in Table 2 and Figure 3.

**Table 2:** A comparison of salivary AST level in healthy and gingivitis groups AST - aspartate aminotransferase

Group	NO	Mean	Std. deviation	Mean. diff	t	Sig. (2-tailed)
Healthy Group	15	18.6	7.7	24.57	6.73	0.00
Gingivitis	15	43.2	11.8			

Statistical analysis revealed significant (t= 12.6, p < 0.01) differences between salivary AST levels in patients with periodontitis compared with the healthy group as shown in Table 3 and Figure 3.

**Table 3:** A comparison of salivary AST level in healthy and periodontitis groups AST - aspartate aminotransferase

Group	NO	Mean	Std. Deviation	Mean. diff	t	Sig. (2-tailed)
Healthy Group	15	18.6	7.7	67.7	12.6	0.00
Periodontitis	15	86.4	19.2			

The results show that severe gingival inflammation and suppuration were related to the highest AST levels.

## Discussion

A specific diagnosis of periodontal tissue that is at risk or has a disease has been a challenge for clinicians [9]. Recent studies focused on investigations that have the absolute assurance to investigate periodontal diseases precisely. It is critical for clinicians today to make reasonable decisions regarding the treatment and prevention of periodontal diseases [10].

Plaque and gingival indexes, bleeding on probing, pocket depth, and attachment loss have been known as traditional methods for diagnosing the periodontium diseases. Still, they focus more on the disease severity more than its activity. So, more recent methods have been expected to assist the diagnosis of periodontal disease activity and provide information about tissues at risk to develop a new disease [7,11].

In medicine, biochemical tests are significantly used both in relation to diseases and those in which biochemical changes result in such a disease and with an obvious metabolic basis. In dentistry, these tests have become more beneficial and crucial in diagnosing, monitoring, prognosis, and screening periodontal diseases that have enzymatic activity changes in the metabolic shift in periodontium [7].

Saliva is an excellent diagnostic tool because it is easy to collect and contains locally and systemically derived biomarkers that help detect periodontal diseases. Multiple studies suggest that saliva is recently used as a diagnostic tool for early detection of periodontitis and gingivitis. There exist a lot of tests using saliva for detecting periodontal diseases but these tests are not utilized regularly now [2].

Many enzymes have been used to assess the progression of periodontal diseases as biomarkers. AST enzyme is one of the biomarkers [3]. AST is a transaminase enzyme that catalyzes the transformation of aspartate and alpha-ketoglutarate to oxaloacetate and glutamate. its greatest concentrations are observed in hepatocytes, making them significant markers of liver pathology. Nevertheless, it is not limited only to the liver. It is found in the heart, kidneys, erythrocyte and skeletal muscle, among other places. This gives it the potential to suggest pathology related to any cell that contains it. The reference value for this enzyme is 12-38 U/L. Elevated AST levels are regularly related to inflammatory liver disease (viral hepatitis), alcoholic liver disease, cirrhosis, cholestatic syndromes, acute myocardial infarction, septic shock. AST levels are also significantly increased in acute pancreatitis, acute renal disease and pulmonary embolism. Pregnancy may induce slight increases in the plasma activity of aminotransferases such as AST [12-15]. Skeletomuscular disorders such as idiopathic inflammatory myopathies, rhabdomyolysis, dermatomyositis, polymyositis and inclusion body myositis can lead to an increased level of AST [16,17]. Furthermore, the AST level is raised due to drug toxicity which is mainly intrinsic and idiosyncratic types. More than 1,000 medications can cause drug toxicity. Most common drugs such as amoxicillin-clavulanate, isoniazid, flutamide, ibuprofen, azathioprine, nitrofurantoin and infliximab [18-20]. Since AST is contained in the cytoplasm of cells and released on cell death, enzyme activity’s level is correlated with active tissue destruction of the periodontium. Thus, such a correlation indicates that AST level can be helpful as a diagnostic tool for assessing periodontal diseases [3].

The present study was designed to assess the relationship between chronic periodontal diseases and biochemical parameters.

All female patients were excluded in this study to prevent the impaction of hormones. Specifically, estrogens can influence the cytodifferentiation of the stratified squamous epithelium as well as the synthesis and maintenance of fibrous collagen. Estrogen receptors found in osteoblast-like cells provide a mechanism for direct action on bone. This hormone may alter immunologic factors and responses, including antigen expression and presentation, cytokine production as well as the expression of apoptotic factors and cell death [21]. Based on in vitro data from cell lines and primary cell cultures, various genes have been suggested over the years as putative targets of the antiresorptive impact of estrogens on bone. This extended list involves cytokines such as interleukin (IL)-1β, IL-6, IL-7, tumor necrosis factor (TNF)-α, M-CSF, RANKL, and OPG formed by bone marrow stromal cells, T and B lymphocytes, macrophages, and dendritic cells. The IL-1 receptor, c-jun, c-fos, cathepsins, and TRAP expressed in osteoclasts have also been associated [22].

Estrogen effects on osteoclasts are believed to be mediated indirectly through non-osteoclastic cells. Loss of estrogen is associated with increased secretion of IL-1, IL-6, and TNF-α from the peripheral blood monocytes, bone marrow stromal cells, or osteoblasts, and reduced expression of TGF-β in bone. Raised levels of these factors result in increased osteoclastogenesis. Furthermore, recently reported that estrogen promoted apoptosis of murine osteoclast-like cells mediated by TGF-β in a mixed cell population in culture. Also, the indirect effects of estrogen on bone resorption facilitated by soluble factors released from non-osteoclastic cells and cell-to-cell contact with non-osteoclastic cells, estrogen can act directly on osteoclasts to limit their bone-resorbing action. The capability of estrogen to stimulate apoptosis of osteoclasts at correlative concentrations effective for inhibition of bone resorption may be the mechanism underlying such effects [23].

Individuals who have taken antibiotics in the past six months or undergone a periodontal treatment in the past six months were also excluded due to the impact of these in enzyme activity. Patients with a history of smoking and alcohol abuse were also excluded because alcohol and smoking constitute a major risk factor for periodontal diseases.

AST is an enzyme that can normally be found confined to the cell. After cell death takes place, it is released into the GCF and saliva during the activeness of periodontal destruction and active gingival inflammation. So, this shows a relationship between the increasing activity of AST and the presence of periodontal destruction [2].

In this study, we assessed the relationship of AST on periodontitis and gingivitis and compared these groups with a control healthy group in 45 samples. Statistical analysis revealed significant (p < 0.01) differences between salivary AST levels in the study groups compared with the control group.

The result of this study demonstrated a significantly increased level of AST enzyme in saliva from patients with periodontitis (Mean=86.4) compared to the healthy group (Mean=18.6), which was significant (p < 0.01). These results agreed with the studies [2,3,11] where AST level in periodontitis patients and healthy patients were studied and showed significant (p < 0.001) [3,11] and (p < 0.05) [2]*.*

The result of this study demonstrated a significantly increased level of AST enzyme in saliva from patients with gingivitis (Mean=43.2) compared to the healthy group (Mean=18.6), which was significant (p < 0.01). These results agreed with the works [3,11] where AST level in periodontitis patients was studied and healthy patients and showed significant (p < 0.001).

## Conclusions

Nowadays, it is essential to make reasonable and cost-effective decisions regarding diagnosing, preventing, and treating periodontal tissue diseases. Treatment and prevention must be based on accurate diagnosis.

Diagnosis of periodontal diseases is based on clinical signs such as a change in color, clinical attachment loss, and bleeding on probing. In addition, radiographic assessment is used as an additional diagnostic tool to confirm the diagnosis. However, these methods are only assessing the disease severity more than its activity. Accurate diagnostic procedures are essential for evaluating the disease activity and monitoring the tissue’s response to the therapy

An evaluation of salivary biomarkers provides a reasonable manner for diagnosing and preventing periodontal diseases. The present study revealed that an increased AST level in the saliva relates to periodontal tissue destruction. This also showed that a patient with gingivitis and periodontitis had a high AST level which indicated clinically that salivary biomarkers could be used as a diagnostic tool for the evaluation of periodontal health status.

In our study, the salivary AST levels are more in patients with periodontitis than patients affected by gingivitis. This is due to an increased release of intercellular enzymes into the saliva from the diseased periodontal tissues. It was also proven the association between the enzyme activity and clinical parameters.
